# Detection of 12 Common Food-Borne Bacterial Pathogens by TaqMan Real-Time PCR Using a Single Set of Reaction Conditions

**DOI:** 10.3389/fmicb.2019.00222

**Published:** 2019-02-13

**Authors:** Ying Liu, Yang Cao, Tao Wang, Qingyang Dong, Junwen Li, Chao Niu

**Affiliations:** Department of Environmental Medicine, Tianjin Institute of Environmental and Operational Medicine, Tianjin, China

**Keywords:** food-borne bacterial pathogens, detection, TaqMan real-time quantitative PCR, virulence gene, meat

## Abstract

Food safety has become an important public health issue worldwide. However, conventional methods for detection of food-borne pathogens are complicated, and labor-intensive. Moreover, the sensitivity is often low, and it is difficult to achieve high-throughput detection. This study developed a TaqMan real-time polymerase chain reaction (PCR) assay for the simultaneous detection and quantification of 12 common pathogens in a single reaction, including *Escherichia coli O157:H7, Listeria monocytogenes/ivanovii, Salmonella enterica, Vibrio parahaemolyticus*, β*-streptococcus* hemolyticus, *Yersinia enterocolitica, Enterococcus faecalis, Shigella* spp., *Proteus mirabilis, Vibrio fluvialis, Staphylococcus aureus*, and *Campylobacter jejuni* in food and drinking water. Based on published sequence data, specific primers, and fluorescently-labeled hybridization probes were designed targeting based on the virulence genes of the 12 pathogens, and these primers and probes were optimized to achieve consistent reaction conditions. The assay was evaluated using 106 pure bacterial culture strains. There was no cross-reaction among the different pathogens. The analytical sensitivity was 1 copy/μL for *E. coli O157:H7, L. monocytogenes/ivanovii*, β*-streptococcus hemolyticus, Shigella* spp., *P. mirabilis*, and *V. fluvialis*, 10 copies/μL for *S. enterica, V. parahaemolyticus, Y. enterocolitica, E. faecalis, S. aureus*, and *C. jejuni*, respectively. The limit of detection (LOD) was 296, 500, 177, 56, 960, 830, 625, 520, 573, 161, 875, and 495 CFU/mL for *E. coli O157:H7, L. monocytogenes/ivanovii, S. enterica, V. parahaemolyticus*, β*-streptococcus hemolyticus, Y. enterocolitica, E. faecalis, Shigella* spp., *P. mirabilis, V. fluvialis, S. aureus*, and *C. jejuni*, respectively. The limit of detection for the assay in meat samples was 10^3^ CFU/g for *V. parahaemolyticus* and 10^4^ CFU/g for other 11 strains. Together, these results indicate that the optimized TaqMan real-time PCR assay will be useful for routine detection of pathogenic bacteria due to its rapid analysis, low cost, high-throughput, high specificity, and sensitivity.

## Introduction

Researchers have identified more than 250 known food-borne illnesses, most of which are infectious, and caused by a variety of bacteria, followed by viruses and parasites (Mangal et al., 2016). Bacterial food-borne diseases are becoming a growing public health concern for the whole world, especially for the developing countries (Fung et al., 2018). According to the World Health Organization (WHO) estimates, food-borne or water-based diarrhea are responsible for about 2.2 million deaths around the world each year (Johnson, 2011). In China, the situation is more optimistic, one in every 6.5 people is suffering from food-borne disease due to the intake of food contaminated with pathogens. *E. coli O157:H7, L. monocytogenes, S. enterica, S. aureus*, and *C. jejuni* contribute to most of the food-borne outbreaks (Mangal et al., 2016). Besides most outbreaks associated with contaminated foods, contaminated drinking water outbreaks have been reported (Park et al., 2011). Given the globally public health and economic burden due to food-borne illness, it is essential to develop reliable, and rapid methods for pathogen detection.

Conventional culture-based methods are still regarded as the “gold standard” for the identification of pathogenic bacteria, but the technique has several disadvantages. It is time-consuming, labor-intensive, and quantitative analysis is difficult to achieve (Wiemer et al., 2011). In addition, false-negative results may happen because of viable but non-culturable (VBNC) pathogens (Law et al., 2014). Furthermore, traditional cultural methods lack good sensitivity and are not capable of simultaneously detecting multiple food-borne pathogens. Thus a rapid, highly sensitive, and inexpensive detection techniques is required (Espy et al., 2006; Park et al., 2010; Ranjbar et al., 2014a; Valencia-Shelton and Loeffelholz, 2014).

Molecular-based methods included are polymerase chain reaction (PCR), real-time PCR, loop-mediated isothermal amplification (LAMP), and microarray which are used to detect food-borne pathogens widely (Zeng et al., 2016). In contrast to conventional single and multiplex PCR, real-time PCR technology that can monitor the products by measuring the fluorescent signal continuously, is most commonly used as a rapid and reliable tool because of its high sensitivity and specificity. Several fluorescent system such as TaqMan probes have been developed (Law et al., 2014). However, the design of primer/probes was a critical problem in TaqMan real-time PCR assays. Owing to less polymorphic loci in the gene, 16S rRNA sequences cannot be used to distinguish closely related bacteria at the species level such as *Escherichia coli* and *Shigella* spp. (Ohara-Nemoto et al., 1997), and cannot identify bacterial pathogenicity. The virulence genes have been used as target genes for nucleic acid-based assays (Finlay and Falkow, 1997; Wiemer et al., 2011; Van Lint et al., 2015). Namely, *flic, hlyA, rfbE* were selected from *E. coli O157:H7, invA, staG* from *Salmonella* spp., *ail, nuc* from *Staphylococcus aureus* and *foxA* genes from *Yersinia enterocolitica* to design primers and probes in real-time TaqMan PCR assay (Ranjbar et al., 2014b; Wang et al., 2014; Ding et al., 2017; Zhou et al., 2017). Additionally, a simultaneous quantifying detection of *V. parahaemolyticus* and *L. monocytogenes* by TaqMan-based real-time PCR using primers and probes that target *tlh* and *hlyA* genes, respectively, was developed (Zhang et al., 2015). Also, 23 individual TaqMan real-time PCR was developed to detect common food-borne pathogens by using TaqMan probes (Cremonesi et al., 2014).

In this study, the primers and probes were designed based on the specific virulence genes of *E. coli O157:H7, L. monocytogenes/ivanovii, S. enterica, V. parahaemolyticus*, β*-streptococcus hemolyticus, Y. enterocolitica, E. faecalis, Shigella spp., P. mirabilis, V. fluvialis, S. aureus* and *C. jejuni*, and a simple, fast, efficient, and economical real-time PCR method using the TaqMan probe for simultaneous detection and quantification of 12 common pathogenic bacteria was established. This assay was evaluated using genomic DNA from pure culture collection strains.

## Materials and Methods

### Bacterial Strains and Culturing Conditions

*Escherichia coli* spp., *Listeria* spp., *Salmonella enterica, Yersinia enterocolitica, Shigella* spp., *Proteus* spp., and *Staphylococcus* spp. were cultured in tryptic soy broth (TSB; BD, USA) at 37°C. *Vibrio parahaemolyticus* were grown in tryptic soy broth supplemented with 3% NaCl at 37°C. *Streptococcus* spp. were grown on tryptic soy agar (TSA; BD, USA) with 5% sheep blood at 37°C to produce isolated colonies. *Vibrio fluvialis* were grown in 2216E liquid medium (Hope Bio-Technology, Qingdao, China) at 37°C. *Campylobacter jejuni* were cultured on Columbia blood agar under microaerophilic conditions (5% O_2_, 10% CO_2_, and 85% N_2_) at 42°C for 24–48 h. Moreover, 12 bacterial strains were used to determine the specificity and sensitivity of the primer pairs (Table 1), and the strains were cultured on the appropriate growth media and under suitable culture conditions. Following overnight incubation, genomic DNA was extracted from supernatants of collected bacterial cells after centrifugation.

**Table 1:** Primer and TaqMan probe sequencens used in this study.

**Pathogen**	**Target gene**	**Accession number**	**Primer (position)**	**Sequence (5' → 3')**	**Amplicon length (bp)**
*Escherichia coli O157:H7*	*rfbE*	S83460.1	190F	CAACGTGGATTTCATCAA	
			337R	TAGGTATATCGGAAGGAGA	148
			296P	AGCAACCGTTCCATTACTTACAG	
*Listeria monocytogenes/ivanovii*	*prfA*	AJ812222.1	235F	TCGGTTGGCTATTATAATTTAG	
			386R	GCTAGACTGTATGAAACTTG	152
			267P	CGAGCAGGCTACCGCATACG	
*Salmonella enterica*	*hilA*	U25352.1	352F	CAACCTACGACTCATACA	
			543R	GCGTAATTGATCCATGAG	192
			524P	TCAAGAATATCCTTAACACTGCGGC	
*Vibrio parahaemolyticus*	*toxR*	AB029915.1	356F	CAGACTCAAGCTCAATTG	
			439R	GCTCTACGATTGTTTCTAC	84
			389P	CTTCTGATAACAATGACGCCTCTGC	
*Streptococcus pyogenes*	*scpA*	901674	356F	CAGACATTAAAGCAAATACTG	
			439R	TCTGCTATTGTTTCTTCTG	154
			389P	AGAAGACACTCCTGCTACCGAAC	
*Yersinia enterocolitica*	*foxA*	X60447.1	352F	CGGTGATGTGAACAATAC	
			543R	GCCATATAACGCAGAAGA	136
			524P	CATCAATACGCTCAAGGAACCACG	
*Enterococcus faecalis*	*ddl*	KC594681.1	47F	CCCATAGTAAAGGATACATAC	
			146R	CGCTGTGATTTCTTCTTA	100
			108P	CCTGAATGAATTGAACACCATGCCT	
*Shigella spp*.	*ipaH9.8*	AY206445.1	242F	TGCTCAATGTATCATATAATCA	
			435R	GCTGATATTCATAGTCAATAAC	194
			267P	AACTAACCTACCTGAACTGCCTGT	
*Proteus mirabilis*	*ureR*	Z18752.1	69F	CCATCAGATTATGTCATTCAA	
			159R	GAGGAAAATGCAATTTATCTTTA	88
			131P	CACACCCTACCCAACATTCATTTCA	
*Vibrio fluvialis*	*toxR*	AF170885.1	387F	TTCGCAGTCTAAATTTCG	
			510R	TCCACCATATTTTCTTACG	124
			423P	CGATGTGATTGTCAGCACGCC	
*Staphylococcus aureus*	*ebpS*	AF400161.1	868F	CCACATGCCTCTAATAATG	
			1064R	GCGATTTTATTTTCTTTTGTAC	197
			1024P	ATGCCATGCCTCCAAATATCGC	
*Campylobacter jejuni*	*mapA*	X80135.1	75F	TGCTCAAGTTAATCAAATTTC	
			156R	CCCTTTAATCTTTGCTTCA	82
			135P	ACCACCAGGACTTTCACAAGAACT	

### Genomic DNA Extraction

DNA used as template for PCR amplification was isolated from ~0.5–1 mL (1.0–5.0E+09 cells) of a freshly grown (18–24 h) bacterial culture using MiniBEST Bacteria Genomic DNA Extraction Kit (TaKaRa, Japan) according to the manufacturer's instructions. The DNA quantity and purity were measured spectrophotometrically via absorbance measurements at A_260_ and A_280_, as well as visualization on 1% agarose gel. A DNA Isolation Reagent for Meat and Meat Products (TaKaRa, Japan) was used to isolate DNA in meat as recommended by the manufacturer.

### Primers and Probes

Virulence genes of the 12 bacterial pathogens were chosen as target genes whose sequences are available from NCBI and the pathogen-specific virulence factors (VFs) from our previous work were selected in priority (Niu et al., 2013). Furthermore, the pathogen-specific conservative sequence fragments of VFs were obtained by carrying out the designed similarity comparative algorithm approach (unpublished data, Yang Cao and Chao Niu). The 12 sets of different primers and TaqMan probes (FAM labeled) based on the pathogen-specific conservative sequence fragments were designed using the Primer Express 3.0 program (http://frodo.wi.mit.edu). Sequence features of each gene such as regions of high or low GC-content, and size were examined to ensure the same amplification conditions. The *in silico* specificity was also analyzed using nucleotide BLAST (http://blast.ncbi.nlm.nih.gov/) from the GenBank sequence database. The primers and TaqMan probes were synthesized by Sangon Biotech (Shanghai, China). Sequences of the primer and probes used for the TaqMan real-time PCR assay, as well as the amplicon size of target genes are listed in Table 1.

### Assessment of Specificity of the Assay Using the Pure Cultures

Specificity of the assay was assessed with 106 bacterial strains that closely and distantly related to the 12 pathogens (Table 2). All bacterial cultures were inoculated into the growth media and under the appropriate growth conditions. Bacterial DNA was extracted according to the procedure described above and was used as a template in the TaqMan real-time quantitative PCR.

**Table 2:** Tested isolates and results for the real-time PCR.

**Isolates**	**Source**	**Targeted gene loci**											
		***rfbE***	***prfA***	***hilA***	***toxR***	***scpA***	***foxA***	***ddl***	***ipaH***	***ureR***	***toxR***	***ebpS***	***mapA***
***ESCHERICHIA*** **SPP. (*****N*** **= 18)**													
*Escherichia coli O157:H7*	ATCC^a^35150	**+**	**–**	**-**	**–**	**–**	**–**	**–**	**–**	**–**	**–**	**–**	**–**
*Escherichia coli O157:H7*	NCTC^b^12900	**+**	**–**	**–**	**–**	**–**	**–**	**–**	**–**	**–**	**–**	**–**	**–**
*Escherichia coli O157:H7*	CICC^c^21531	**+**	**–**	**–**	**–**	**–**	**–**	**–**	**–**	**–**	**–**	**–**	**–**
*Escherichia coli O157:H7*	Stored in our laboratory	**+**	**–**	**–**	**–**	**–**	**–**	**–**	**–**	**–**	**–**	**–**	**–**
*Escherichia coli O26*	Stored in our laboratory	**–**	**–**	**–**	**–**	**–**	**–**	**–**	**–**	**–**	**–**	**–**	**–**
*Escherichia coli O138*	Stored in our laboratory	**–**	**–**	**–**	**–**	**–**	**–**	**–**	**–**	**–**	**–**	**–**	**–**
*Escherichia coli O139*	Stored in our laboratory	**–**	**–**	**–**	**–**	**–**	**–**	**–**	**–**	**–**	**–**	**–**	**–**
*Escherichia coli*	ATCC13706	**–**	**–**	**–**	**–**	**–**	**–**	**–**	**–**	**–**	**–**	**–**	**–**
*Escherichia coli*	ATCC25922	**–**	**–**	**–**	**–**	**–**	**–**	**–**	**–**	**–**	**–**	**–**	**–**
*Escherichia coli*	CMCC^d^44113	**–**	**–**	**–**	**–**	**–**	**–**	**–**	**–**	**–**	**–**	**–**	**–**
*Escherichia coli*	CMCC44505	**–**	**–**	**–**	**–**	**–**	**–**	**–**	**–**	**–**	**–**	**–**	**–**
*Escherichia coli*	CMCC44216	**–**	**–**	**–**	**–**	**–**	**–**	**–**	**–**	**–**	**–**	**–**	**–**
*Escherichia coli*	CMCC44338	**–**	**–**	**–**	**–**	**–**	**–**	**–**	**–**	**–**	**–**	**–**	**–**
*Escherichia coli*	CMCC44336	**–**	**–**	**–**	**–**	**–**	**–**	**–**	**–**	**–**	**–**	**–**	**–**
*Escherichia coli*	CMCC44110	**–**	**–**	**–**	**–**	**–**	**–**	**–**	**–**	**–**	**–**	**–**	**–**
*Escherichia coli*	CMCC44824	**–**	**–**	**–**	**–**	**–**	**–**	**–**	**–**	**–**	**–**	**–**	**–**
*Escherichia coli*	CMCC44825	**–**	**–**	**–**	**–**	**–**	**–**	**–**	**–**	**–**	**–**	**–**	**–**
*Escherichia coli*	CGMCC^e^1.8732	**–**	**–**	**–**	**–**	**–**	**–**	**–**	**–**	**–**	**–**	**–**	**–**
***LISTERIA*** **SPP. (*****N*** **= 7)**													
*Listeria monocytogenes*	ATCC19118	**–**	**+**	**–**	**–**	**–**	**–**	**–**	**–**	**–**	**–**	**–**	**–**
*Listeria monocytogenes*	CMCC54001	**–**	**+**	**–**	**–**	**–**	**–**	**–**	**–**	**–**	**–**	**–**	**–**
*Listeria monocytogenes*	Stored in our laboratory	**–**	**+**	**–**	**–**	**–**	**–**	**–**	**–**	**–**	**–**	**–**	**–**
*Listeria monocytogenes*	ATCC19115	**–**	**+**	**–**	**–**	**–**	**–**	**–**	**–**	**–**	**–**	**–**	**–**
*Listeria ivanovii*	ATCC19119	**–**	**+**	**–**	**–**	**–**	**–**	**–**	**–**	**–**	**–**	**–**	**–**
*Listeria ivanovii*	C4(20031122)	**–**	**+**	**–**	**–**	**–**	**–**	**–**	**–**	**–**	**–**	**–**	**–**
*Listeria welshimeri*	GDMCC^f^1.232	**–**	**–**	**–**	**–**	**–**	**–**	**–**	**–**	**–**	**–**	**–**	**–**
*Listeria innocua*	ATCC33090	**–**	**–**	**–**	**–**	**–**	**–**	**–**	**–**	**–**	**–**	**–**	**–**
***SALMONELLA*** **SPP. (*****N*** **= 12)**													
*Salmonella enterica* subsp.*enterica*	Stored in our laboratory	**–**	**–**	**+**	**–**	**–**	**–**	**–**	**–**	**–**	**–**	**–**	**–**
*Salmonella enterica* subsp.*enterica*	ATCC14028	**–**	**–**	**+**	**–**	**–**	**–**	**–**	**–**	**–**	**–**	**–**	**–**
*Salmonella enterica* serovar Choleraesuis	CMCC50306	**–**	**–**	**+**	**–**	**–**	**–**	**–**	**–**	**–**	**–**	**–**	**–**
*Salmonella enterica* serovar Typhimurium	CMCC50115	**–**	**–**	**+**	**–**	**–**	**–**	**–**	**–**	**–**	**–**	**–**	**–**
*Salmonella enterica* serovar Senftenberg	CMCC50315	**–**	**–**	**+**	**–**	**–**	**–**	**–**	**–**	**–**	**–**	**–**	**–**
*Salmonella enterica* serovar Aberdeen	CMCC50312	**–**	**–**	**+**	**–**	**–**	**–**	**–**	**–**	**–**	**–**	**–**	**–**
*Salmonella enteria* serovar Aberdeen	CMCC50313	**–**	**–**	**+**	**–**	**–**	**–**	**–**	**–**	**–**	**–**	**–**	**–**
*Salmonella enterica* serovar Paratyphi	CMCC50774	**–**	**–**	**+**	**–**	**–**	**–**	**–**	**–**	**–**	**–**	**–**	**–**
*Salmonella enterica* serovar Rubislaw	CMCC50798	**–**	**–**	**+**	**–**	**–**	**–**	**–**	**–**	**–**	**–**	**–**	**–**
*Salmonella entericca* serovar Champaign	CMCC50067	**–**	**–**	**+**	**–**	**–**	**–**	**–**	**–**	**–**	**–**	**–**	**–**
*Salmonella enterica* serovar Paratyphi A	CMCC50093	**–**	**–**	**+**	**–**	**–**	**–**	**–**	**–**	**–**	**–**	**–**	**–**
*Salmonella enterica* serovar Paratyphi B	CMCC50094	**–**	**–**	**+**	**–**	**–**	**–**	**–**	**–**	**–**	**–**	**–**	**–**
***VIBRIO*** **SPP. (*****N*** **= 12)**													
*Vibrio fluvialis*	ATCC33812	**–**	**–**	**–**	**+**	**–**	**–**	**–**	**–**	**–**	**–**	**–**	**–**
*Vibrio fluvialis*	ATCC33809	**–**	**–**	**–**	**+**	**–**	**–**	**–**	**–**	**–**	**–**	**–**	**–**
*Vibrio fluvialis*	ATCC33810	**–**	**–**	**–**	**+**	**–**	**–**	**–**	**–**	**–**	**–**	**–**	**–**
*Vibrio fluvialis*	CGMCC1.1610	**–**	**–**	**–**	**+**	**–**	**–**	**–**	**–**	**–**	**–**	**–**	**–**
*Vibrio parahaemolyticus*	ATCC17802	**–**	**–**	**–**	**–**	**–**	**–**	**–**	**–**	**–**	**+**	**–**	**–**
*Vibrio parahaemolyticus*	CMCC20502	**–**	**–**	**–**	**–**	**–**	**–**	**–**	**–**	**–**	**+**	**–**	**–**
*Vibrio parahaemolyticus*	CMCC20516	**–**	**–**	**–**	**–**	**–**	**–**	**–**	**–**	**–**	**+**	**–**	**–**
*Vibrio alginolyticus*	ATCC17749	**–**	**–**	**–**	**–**	**–**	**–**	**–**	**–**	**–**	**–**	**–**	**–**
*Vibrio vulnificus*	ATCC27562	**–**	**–**	**–**	**–**	**–**	**–**	**–**	**–**	**–**	**–**	**–**	**–**
*Vibrio vulnificus*	CGMCC1.8674	**–**	**–**	**–**	**–**	**–**	**–**	**–**	**–**	**–**	**–**	**–**	**–**
*Vibrio cholerae*	GIM1.449	**–**	**–**	**–**	**–**	**–**	**–**	**–**	**–**	**–**	**–**	**–**	**–**
*vibrio proteolyticus*	ATCC15338	**–**	**–**	**–**	**–**	**–**	**–**	**–**	**–**	**–**	**–**	**–**	**–**
***STREPTOCOCCUS*** **SPP. (*****N*** **= 5)**													
*Streptococcus pyogenes*	ATCC19615	**–**	**–**	**–**	**–**	**+**	**–**	**–**	**–**	**–**	**–**	**–**	**–**
*β-Hemolytic streptococcus*	CMCC32210	**–**	**–**	**–**	**–**	**+**	**–**	**–**	**–**	**–**	**–**	**–**	**–**
*β-Hemolytic streptococcus*	CMCC32204	**–**	**–**	**–**	**–**	**+**	**–**	**–**	**–**	**–**	**–**	**–**	**–**
*Streptococcus pneumoniae*	ATCC49619	**–**	**–**	**–**	**–**	**–**	**–**	**–**	**–**	**–**	**–**	**–**	**–**
*Streptococcus thermophilus*	CGMCC1.6472	**–**	**–**	**–**	**–**	**–**	**–**	**–**	**–**	**–**	**–**	**–**	**–**
***YERSINIA*** **SPP. (*****N*** **= 9)**													
*Yersinia enterocolitica*	CMCC52225	**–**	**–**	**–**	**–**	**–**	**+**	**–**	**–**	**–**	**–**	**–**	**–**
*Yersinia enterocolitica*	CMCC52219	**–**	**–**	**–**	**–**	**–**	**+**	**–**	**–**	**–**	**–**	**–**	**–**
*Yersinia enterocolitica*	CMCC52301	**–**	**–**	**–**	**–**	**–**	**+**	**–**	**–**	**–**	**–**	**–**	**–**
*Yersinia enterocolitica*	CMCC52302	**–**	**–**	**–**	**–**	**–**	**+**	**–**	**–**	**–**	**–**	**–**	**–**
*Yersinia enterocolitica*	CMCC52203	**–**	**–**	**–**	**–**	**–**	**+**	**–**	**–**	**–**	**–**	**–**	**–**
*Yersinia enterocolitica*	CMCC52206	**–**	**–**	**–**	**–**	**–**	**+**	**–**	**–**	**–**	**–**	**–**	**–**
*Yersinia enterocolitica*	ATCC23715	**–**	**–**	**–**	**–**	**–**	**+**	**–**	**–**	**–**	**–**	**–**	**–**
*Yersinia pseudotuberculosis*	CMCC53504	**–**	**–**	**–**	**–**	**–**	**–**	**–**	**–**	**–**	**–**	**–**	**–**
*Yersinia intermedia*	CGMCC1.6197	**–**	**–**	**–**	**–**	**–**	**–**	**–**	**–**	**–**	**–**	**–**	**–**
***ENTEROCOCCUS*** **SPP. (*****N*** **= 5)**													
*Enterococcus faecalis*	ATCC19433	**–**	**–**	**–**	**–**	**–**	**–**	**+**	**–**	**–**	**–**	**–**	**–**
*Enterococcus faecalis*	ATCC29212	**–**	**–**	**–**	**–**	**–**	**–**	**+**	**–**	**–**	**–**	**–**	**–**
*Enterococcus faecalis*	CMCC32001	**–**	**–**	**–**	**–**	**–**	**–**	**+**	**–**	**–**	**–**	**–**	**–**
*Enterococcus faecalis*	CICC20419	**–**	**–**	**–**	**–**	**–**	**–**	**+**	**–**	**–**	**–**	**–**	**–**
*Enterococcus faecalis*	ATCC700802	**–**	**–**	**–**	**–**	**–**	**–**	**+**	**–**	**–**	**–**	**–**	**–**
***SHIGELLA*** **SPP. (*****N*** **= 15)**													
*Shigella spp*.	ATCC12038	**–**	**–**	**–**	**–**	**–**	**–**	**–**	**+**	**–**	**–**	**–**	**–**
*Shigella flexneri*	ATCC12022	**–**	**–**	**–**	**–**	**–**	**–**	**–**	**+**	**–**	**–**	**–**	**–**
*Shigella flexneri*	CMCC51066	**–**	**–**	**–**	**–**	**–**	**–**	**–**	**+**	**–**	**–**	**–**	**–**
*Shigella flexneri*	CMCC51571	**–**	**–**	**–**	**–**	**–**	**–**	**–**	**+**	**–**	**–**	**–**	**–**
*Shigella flexneri*	CMCC51508	**–**	**–**	**–**	**–**	**–**	**–**	**–**	**+**	**–**	**–**	**–**	**–**
*Shigella flexneri*	ATCC12022	**–**	**–**	**–**	**–**	**–**	**–**	**–**	**+**	**–**	**–**	**–**	**–**
*Shigella flexneri*	CMCC51067	**–**	**–**	**–**	**–**	**–**	**–**	**–**	**+**	**–**	**–**	**–**	**–**
*Shigella dysenteriae*	CMCC51135	**–**	**–**	**–**	**–**	**–**	**–**	**–**	**+**	**–**	**–**	**–**	**–**
*Shigella dysenteriae*	CMCC51336	**–**	**–**	**–**	**–**	**–**	**–**	**–**	**+**	**–**	**–**	**–**	**–**
*Shigella dysenteriae*	CMCC51252	**–**	**–**	**–**	**–**	**–**	**–**	**–**	**+**	**–**	**–**	**–**	**–**
*Shigella sonnei*	CMCC51424	**–**	**–**	**–**	**–**	**–**	**–**	**–**	**+**	**–**	**–**	**–**	**–**
*Shigella sonnei*	CMCC51081	**–**	**–**	**–**	**–**	**–**	**–**	**–**	**+**	**–**	**–**	**–**	**–**
*Shigella sonnei*	ATCC25931	**–**	**–**	**–**	**–**	**–**	**–**	**–**	**+**	**–**	**–**	**–**	**–**
*Shigella boydii*	CMCC51515	**–**	**–**	**–**	**–**	**–**	**–**	**–**	**+**	**–**	**–**	**–**	**–**
*Shigella boydii*	CMCC51510	**–**	**–**	**–**	**–**	**–**	**–**	**–**	**+**	**–**	**–**	**–**	**–**
***PROTEUS*** **SPP. (*****N*** **= 8)**													
*Proteus mirabilis*	ATCC35659	**–**	**–**	**–**	**–**	**–**	**–**	**–**	**–**	**+**	**–**	**–**	**–**
*Proteus mirabilis*	CMCC49005	**–**	**–**	**–**	**–**	**–**	**–**	**–**	**–**	**+**	**–**	**–**	**–**
*Proteus vulgaris*	CMCC49027	**–**	**–**	**–**	**–**	**–**	**–**	**–**	**–**	**–**	**–**	**–**	**–**
*Proteus vulgaris*	ACCC^g^11002	**–**	**–**	**–**	**–**	**–**	**–**	**–**	**–**	**–**	**–**	**–**	**–**
*Proteus vulgaris*	CMCC49001	**–**	**–**	**–**	**–**	**–**	**–**	**–**	**–**	**–**	**–**	**–**	**–**
*Proteus vulgaris*	CMCC49107	**–**	**–**	**–**	**–**	**–**	**–**	**–**	**–**	**–**	**–**	**–**	**–**
*Proteus vulgaris*	CMCC49101	**–**	**–**	**–**	**–**	**–**	**–**	**–**	**–**	**–**	**–**	**–**	**–**
*Proteus penneri*	ATCC33519	**–**	**–**	**–**	**–**	**–**	**–**	**–**	**–**	**–**	**–**	**–**	**–**
***STAPHYLOCOCCUS*** **SPP. (*****N*** **= 7)**													
*Staphylococcus aureus*	Stored in our laboratory	**–**	**–**	**–**	**–**	**–**	**–**	**–**	**–**	**–**	**–**	**+**	**–**
*Staphylococcus aureus*	ATCC43300	**–**	**–**	**–**	**–**	**–**	**–**	**–**	**–**	**–**	**–**	**+**	**–**
*Staphylococcus aureus*	ATCC29213	**–**	**–**	**–**	**–**	**–**	**–**	**–**	**–**	**–**	**–**	**+**	**–**
*Staphylococcus aureus*	ATCC27217	**–**	**–**	**–**	**–**	**–**	**–**	**–**	**–**	**–**	**–**	**+**	**–**
*Staphylococcus aureus*	ATCC6538	**–**	**–**	**–**	**–**	**–**	**–**	**–**	**–**	**–**	**–**	**+**	**–**
*Staphylococcus epidermidis*	ATCC14990	**–**	**–**	**–**	**–**	**–**	**–**	**–**	**–**	**–**	**–**	**–**	**–**
*Staphylococcus epidermidis*	ATCC49134	**–**	**–**	**–**	**–**	**–**	**–**	**–**	**–**	**–**	**–**	**–**	**–**
***CAMPYLOBACTER*** **SPP. (*****N*** **= 2)**													
*Campylobacter jejuni*	ATCC33252	**–**	**–**	**–**	**–**	**–**	**–**	**–**	**–**	**–**	**–**	**–**	**+**
*Campylobacter jejuni*	ATCCBAA−1153	**–**	**–**	**–**	**–**	**–**	**–**	**–**	**–**	**–**	**–**	**–**	**+**
**OTHER (*****N*** **= 5)**													
*Klebsiella peneumoniae*	CMCC46117	**–**	**–**	**–**	**–**	**–**	**–**	**–**	**–**	**–**	**–**	**–**	**–**
*Enterobacter Sakazakii*	CMCC45401	**–**	**–**	**–**	**–**	**–**	**–**	**–**	**–**	**–**	**–**	**–**	**–**
*Bacillus cereus*	CMCC63302	**–**	**–**	**–**	**–**	**–**	**–**	**–**	**–**	**–**	**–**	**–**	**–**
*Pseudomonas aeruginosa*	ATCC9027	**–**	**–**	**–**	**–**	**–**	**–**	**–**	**–**	**–**	**–**	**–**	**–**
*Clostridium perfringens*	ATCC13124	**–**	**–**	**–**	**–**	**–**	**–**	**–**	**–**	**–**	**–**	**–**	**–**

a *ATCC, American Type Culture Collection, USA*;

b *NCTC, National Collection of Type Cultures, U.K.*;

c *CICC, China Center of Industrial Culture Collection*;

d *CMCC, China Medical Culture Collection*;

e *CGMCC, China General Microbiological Culture Collection Center*;

f *GDMCC, Guangdong Microbial Culture Center*;

g *ACCC, Agricultural Culture Collection of China*.

### Construction of Standard Plasmid and Standard Curve

Standard plasmids were constructed using clonal transformation. All purified fragment was cloned into the PMD19-T vector, and then transformed into E. coli strain DH5α. Plasmids constructed were isolated from cultures using the Plasmid Mini Kit (Omega, USA) according to the manufactures instruction. Purity and concentration of the DNA were checked using a Nanodrop-2000 Spectrophotometer (NanoDrop Technologies, USA). The copy number were calculated according to the formula:

$$\begin{array}{l} \frac{6.02^{*}10^{23}*\left(ng/\mu L^{*}10^{-9}\right)}{bp^{*}660} \end{array}$$

Plasmid DNA containing the target amplicon was diluted to contain 10^0^-10^6^ copies/μL and used as the plasmid standard series. Linear relationship between Ct (threshold cycle) and log input DNA copies was established using the real-time PCR assay. The amplification efficiencies (E) was calculated by using the slope of standard curve and applying the following equation:

$$\begin{array}{l} E=10^{-1/slope}-1 \end{array}$$

### TaqMan Real-Time Quantitative PCR

ABI 7500 FAST Cycler was used to carry out a real-time PCR. Amplification reactions were performed in a 25 μL reaction volume involving of 12.5 μL Premix Ex Taq™ (Probe qPCR, contains TaKaRa Ex Taq HS, dNTP Mixture, Mg^2+^, Tli RnaseH, Japan), 1 μL of each primer (10 μM), 1 μL of probe (5 μM), 1 μL of genomic DNA template, and 8.5 μL of H_2_O. Template DNA used in the designed assays is from target and non-target related strains. Each sample was tested in duplicate. The optimized PCR reaction conditions was 95°C for 30 s, followed by 40 cycles of 95°C for 5 s, 55°C for 10 s, and 72°C for 30 s. During the extension phase, fluorescence intensity was measured and the data were analyzed using 7500 Software, Version 2.0.6. For all PCR assays, positive controls were run along with a negative control, which consisted of sterile water. The test result was considered to be positive when Ct value was 36 or less.

### Data Analysis

Data analysis was performed by Software of ABI 7500 FAST (7500 Software, Version 2.0.6). The Ct value is the cycle number at which the amplification curve crosses the fluorescence threshold. Within the exponential phase of the run, the threshold is set manually. A signal for non-template control (NTC) was set at 15 %, thus only an increase in fluorescence intensity of at least 15 %, was considered a positive result.

### Sensitivity Assays

The minimum amount of template DNA (copies/μL) was determined using a series of 10-fold dilutions of plasmid DNA containing the target amplicon by the TaqMan real-time PCR assay. Each reaction was amplified in duplicate. There is a linear relationship that the different template DNA concentrations (log10 copies/μL) were plotted against the corresponding Ct values.

### Evaluation of the Limit of Detection (LOD)

Reference strains cultured overnight with gentle shaking (220 rpm/min) were serially 10-fold diluted in 0.9% NaCl during the logarithmic growth phase. Each dilution (1 mL) of bacterial culture was incubated in order to determine its concentration by plate counts in triplicate (CFU/mL). Then the dilutions were used for DNA extraction as described above followed by absolute quantitative real-time PCR.

### Spiked Food Matrices

The bacterial cultures were diluted to the desired concentration with sterile phosphate buffered saline (PBS) (from 10^7^ to 10^1^ CFU/mL). The precise number of CFU in the dilutions was determined by plate counting method. The serial dilution concentrations of 12 available pure cultures were spiked onto 25 g of fresh minced meat purchased from a local super market. The samples were mechanically homogenized with a stomacher blender in 225 mL buffered peptone water (BPW; Merck, Germany). Non-inoculated food was subjected to the same procedure and used as a negative control. The food products with bacterial solution concentrations ranging from 10^7^ to 10^1^ CFU/g were concentrated, and the DNA from 1 mL homogenate was extracted using the method described above used as a template in real-time PCR experiments.

## Results

### Probe Design

A BLAST search of GenBank *in silico* was used to test each TaqMan assay which revealed no identical sequences other than those targeted. The TaqMan real-time PCR assays developed in this study were performed under uniform conditions. Among the 12 bacteria cultures (106 strains), none of the non-targets were amplified in the detection assays. In addition, non-target bacterial species such as *Klebsiella peneumoniae, Enterobacter Sakazakii, Bacillus cereus, Pseudomonas aeruginosa*, and *Clostridium perfringens* did not produce an amplified signal.

### Genomic DNA Extraction

The extracted DNA from 106 strains by TaKaRa Genomic DNA extraction kit yielded DNA concentrations ranging from 20 to 150 ng/μL with a mean λ_260/280_ of 1.77 ± 0.09.

### Analytical Specificity

Based on the analysis of 106 bacterial strains, the TaqMan real-time PCR was 100% specific (Table 2). No false positive or negative results were found in the established TaqMan assays, confirming the exclusivity.

### Standard Curve and Analytical Sensitivity

Correlation coefficients (*R*^2^) of standard curves constructed were >0.99 and the reaction efficiencies ranged from 92 to 105% for the genes, indicating high linearity. As reported in Table 3, the analytical sensitivity was 1 copies/μL for *E. coli O157:H7, L. monocytogenes/ivanovii*, β*-streptococcus hemolyticus, Shigella* spp., *P. mirabilis, and V. fluvialis*, and 10 copies/μL for *S. enterica, V. parahaemolyticus, Y. enterocolitica, E. faecalis, S. aureus*, and *C. jejuni*. The full regression lines are reported in Table 3.

**Table 3:** Sensitivity and efficiency of the TaqMan real-time PCR obtained by series of 10-fold dilutions of the Standard plasmid.

	**Linear regression line**	***R^**2**^***	**Eff%**	**Sensitivity (copies/μL)**
*Escherichia coli O157:H7*	Y = −3.255lgX + 37.064	0.999	102.888	1
*Listeria monocytogenes*	Y = −3.361lgX + 39.55	0.998	98.383	1
*Salmonella enterica*	Y = −3.283lgX + 40.137	0.999	101.638	10
*Vibrio parahaemolyticus*	Y = −3.203lgX + 38.696	0.999	105.219	10
*β-streptococcus hemolyticus*	Y = −3.51lgX + 40.438	0.998	92.697	1
*Yersinia enterocolitica*	Y = −3.351lgX + 39.435	0.999	98.797	10
*Enterococcus faecalis*	Y = −3.388lgX + 40.01	1	97.311	10
*Shigella spp*.	Y = −3.357lgX + 40.634	0.998	98.573	1
*Proteus mirabilis*	Y = −3.327lgX + 39.762	1	99.785	1
*Vibrio fluvialis*	Y = −3.434lgX + 38.893	0.999	95.53	1
*Staphylococcus aureu*	Y = −3.459lgX + 38.972	0.998	94.596	10
*Campylobacter jejun*	Y = −3.527lgX + 41.121	0.998	92.114	10

### Limit of Detection and Sensitivity in Spiked Food Matrices

Based on the concentration of 12 bacteria by plate counting (CFU/mL) and absolute copy numbers (copies/μL) determined from the corresponding standard curves, according to the analytical sensitivity results (copies/μL), LODs (limit of detection) of the TaqMan real-time PCR assays were equivalent to 296, 500, 177, 56, 960, 830, 625, 520, 573, 161, 875, and 495 CFU/mL for *E. coli O157:H7, L. monocytogenes/ivanovii, S.enterica, V. parahaemolyticus*, β*-streptococcus hemolyticus, Y. enterocolitica, E. faecalis, Shigella* spp., *P. mirabilis, V. fluvialis, S. aureus*, and *C. jejuni*, respectively.

The ability of all the TaqMan assays to detect 12 strains in spiked food samples was tested using artificial spiked serial dilutions of each pathogen into meat. The TaqMan assays revealed good amplification without inhibition of the amplification. *V. parahaemolyticus* was detected in spiked samples in the range of 10^3^-10^7^ CFU/g, and the other 11 strains were detected in concentrations ranging from 10^4^ to 10^7^ CFU/g.

## Discussion

Culture-based methods are not sufficient to detect and prevent all outbreaks of food-borne illness. Methods for rapid, sensitive, and specific detection of food-borne pathogens are required. Nucleic acid-based methods such as PCR and loop-mediated isothermal amplification (LAMP) methods have advantages in sensitivity, specificity, and speed. Among these molecular methods, real-time PCR represents a powerful tool that is more sensitive and suitable for high-throughput analysis (Kralik and Ricchi, 2017). The major advantage of real-time PCR is that less time is required during the analysis. However, it could be affected by PCR inhibitors exist in DNA extracted from food matrix (Vital et al., 2017). In fact, in the analysis process DNA extraction is the first step and high-quality DNA is the most important element to assure subsequent real-time PCR (Cremonesi et al., 2014). For complex sample matrices, automated extraction methods do not reduce the yield of target DNA compared to well-established column-based extraction. The combination of the two methods may ensure reproducibility of the analysis and improve accuracy (Frickmann et al., 2015).

In this study, we established TaqMan real-time PCR assays to simultaneously detect 12 specific food-borne bacterial pathogens including *E. coli O157:H7, L. monocytogenes/ivanovii, S. enterica, V. parahaemolyticus*, β*-streptococcus hemolyticus, Y. enterocolitica, E. faecalis, Shigella* spp., *P. mirabilis, V. fluvialis, S. aureus*, and *C. jejuni*. The assays showed strong strain specificity and exclusivity (Table 2). The results of cultured bacteria and spiked food samples exhibited highly efficient identification. Moreover, the manual protocol reduced the analysis time to ~1 h, compared with the 7 days or more required for the conventional culture-based methods.

Primers and probes ensure sensitive, specific, and simultaneous detection through a single set of amplification conditions. Although 16S rRNA gene has been used for identification of bacteria at the species level, there still are some limitations. The 16S rRNA gene may not be sufficiently discriminative for species differentiation, and closely related bacteria cannot be discerned because of its low rate of base substitutions (Ruan et al., 2017). Although the homology of the genomes of various genus pathogens is very high and there is no difference in the biochemical reaction between the non-pathogenic types of the same genus, the specificity of virulence genes related to pathogenicity is high. In this investigation, specific primers and probes were designed targeting bacterial virulence genes to differentiate the closely related species. Then primers and probes were optimized based on the Tm and GC content to perform experiments under the same reaction conditions. Although TaqMan probes are more costly than SYBR green, the use of the sequence specific probes ensures high sensitivity, and specificity (Postollec et al., 2011; Law et al., 2014). Some target genes selected in the present study, as well as functional genes related to virulence or metabolism, have showed good specificity toward *C. jejuni, Shigella* spp., and *Y. enterocolitica*, when compared to standard methods (Wiemer et al., 2011; Wang et al., 2014; Van Lint et al., 2015). Three specific genes, namely, *rfbE* from *E. coli O157:H7, hilA* from *S. enterica* and *prfA* from *L. monocytogenes* were selected in our study to design primers and probes while *fliC, invA*, and *hlyA* were used in previous studies (Zhou et al., 2017).

The development of high-throughput qPCR platforms was a breakthrough which result in promising systems that are capable of processing a large number of samples simultaneously and also to perform a large number of assays per sample. The more target genes were amplified in a single set of reaction conditions in these high-throughput qPCR formats, the more pathogens were effectively detected or identified at the same time. A laboratory-developed TaqMan Array Card for simultaneous detection of 19 enteropathogens and high-throughput assay for rapid detection of nine pathogens directly from stools with diarrhea have been reported (Antikainen et al., 2013; Liu et al., 2013). In the work of Ishii et al. microfluidic quantitative PCR (qPCR) technology was applied for the simultaneous detection and quantification of multiple food- and waterborne pathogens as *L. monocytogenes, Salmonella* Typhimurium, *V. parahaemolyticus*, and *Clostridium perfringens* (Ishii et al., 2013). Rapid and high-throughput identification of more food-borne bacterial pathogens by multiple analysis platforms have been developed and combination of several detection methods is also possible (Cremonesi et al., 2014; Salihah et al., 2016; Nasrabadi et al., 2017; Thomas et al., 2017; Ahn et al., 2018; Carloni et al., 2018; Zhang et al., 2018).

In conclusion, real-time PCR assays offer the possibility of rapid detection of pathogenic bacteria with higher specificity, sensitivity, and reliability than traditional culture methods. We developed high-throughput qPCR assays that can be run in identical PCR conditions allow the inclusion of large number of assays and samples in one run. These assays enable processing of several samples simultaneously through high-throughput screening of multiple pathogens that brings great saving of time. In summary, the TaqMan real-time PCR assays performed well in contrast with conventional culture-based methods, indicating that it could be an rapid and effective alternative for the identification and diagnosis of food-borne outbreaks.

## Author Contributions

CN and JL conceived and designed the experiments. YL, QD, TW, and CN performed the experiments. YC and CN designed the probes and primers, and analyzed the data. YL, YC, CN, and JL wrote the manuscript. All authors read and approved the final manuscript.

### Conflict of Interest Statement

The authors declare that the research was conducted in the absence of any commercial or financial relationships that could be construed as a potential conflict of interest.
